# Identification of Proteins Sensitive to Thermal Stress in Human Neuroblastoma and Glioma Cell Lines

**DOI:** 10.1371/journal.pone.0049021

**Published:** 2012-11-08

**Authors:** Guilian Xu, Stanley M. Stevens, Firas Kobiessy, Hilda Brown, Scott McClung, Mark S. Gold, David R. Borchelt

**Affiliations:** 1 Department of Neuroscience, Santa Fe Health Alzheimer's Disease Research Center, University of Florida, Gainesville, Florida, United States of America; 2 Department of Cell Biology, Microbiology and Molecular Biology, University of South Florida, Tampa, Florida, United States of America; 3 Department of Psychiatry, University of Florida, Gainesville, Florida, United States of America; 4 Interdisciplinary Center of Biotechnology Research (ICBR), University of Florida, Gainesville, Florida, United States of America; Consejo Superior de Investigaciones Cientificas, Spain

## Abstract

Heat-shock is an acute insult to the mammalian proteome. The sudden elevation in temperature has far-reaching effects on protein metabolism, leads to a rapid inhibition of most protein synthesis, and the induction of protein chaperones. Using heat-shock in cells of neuronal (SH-SY5Y) and glial (CCF-STTG1) lineage, in conjunction with detergent extraction and sedimentation followed by LC-MS/MS proteomic approaches, we sought to identify human proteins that lose solubility upon heat-shock. The two cell lines showed largely overlapping profiles of proteins detected by LC-MS/MS. We identified 58 proteins in detergent insoluble fractions as losing solubility in after heat shock; 10 were common between the 2 cell lines. A subset of the proteins identified by LC-MS/MS was validated by immunoblotting of similarly prepared fractions. Ultimately, we were able to definitively identify 3 proteins as putatively metastable neural proteins; FEN1, CDK1, and TDP-43. We also determined that after heat-shock these cells accumulate insoluble polyubiquitin chains largely linked via lysine 48 (K-48) residues. Collectively, this study identifies human neural proteins that lose solubility upon heat-shock. These proteins may represent components of the human proteome that are vulnerable to misfolding in settings of proteostasis stress.

## Introduction

Recent studies have suggested that a delicate balance of the chaperone network and protein degradation machinery function in concert to maintain the cellular proteome [reviewed by [1]]. The term proteostasis has been used to refer to protein homeostasis, which describes the balance in systems that maintain the proteome. In invertebrate models, the expression of mutant proteins that are aggregation prone can produce a disturbance in the protein homeostasis system, causing broad effects on the folding of cellular proteins [2]. In the C. elegans model system used by Gidalevitz and coworkers, expression of aggregating fragments of mutant huntingtin imposed a burden on protein homeostasis such that co-expressed temperature sensitive mutant proteins failed to achieve active conformations [3]. In this model, the temperature sensitive proteins were thought to be inherently metastable, meaning that at physiologic temperatures these proteins sample conformations that are fully or partially disordered.

In the present study, we sought to identify neural proteins that are sensitive to thermal denaturation upon moderate heat-shock. Heat shock may be viewed as an acute insult to proteostasis that produces far reaching disturbances in the protein homeostasis network. Increasing temperature is assumed to cause an accumulation of misfolded proteins, triggering activation of the ubiquitin-proteasome pathway [4], [5] and inducing the expression of molecular chaperones [6]. We used a moderate heat-shock insult in two neural cell lines, neuroblastoma SH-SY5Y and astrocytoma CCF-STTG1, as a model system to develop protocols to detect the molecular signatures of disturbances in protein homeostasis. Thermal denaturation exposes hydrophobic surfaces within vulnerable proteins, causing a cascade of aberrant protein-protein interactions that lead to the formation of large, heterogeneous, insoluble protein aggregates. Detergent extraction and centrifugation sedimentation were used to separate well-folded from misfolded proteins as a result of heat-denaturation. Using LC-MS/MS approaches, we identified 37 proteins in SH-SY5Y cells and 31 proteins in STTG-1 cells representing multiple functional categories that were susceptible to thermal destabilization. Heat-shocked cells also accumulated high levels of lysine 48 (K-48) linked polyubiquitin. The proteins that lose solubility upon heat-shock may represent natural metastable proteins that could be used as biomarkers to probe the integrity of the protein homeostasis network.

## Methods

### Cell Culture and Heat-shock Treatment

SH-SY5Y [CRL-2266™, American Type Culture Collection (ATCC), Rockville, MD, USA] and CCF-STTG1 (CRL-1718TM, ATCC) cell lines were used in this study. SH-SY5Y cells (3–4 passages from frozen seed stock) were cultured in Dulbecco’s Modified Eagle Medium (DMEM) with 10% fetal bovine serum (FBS) and 4 mM L-glutamine at 37°C in 100 mm dishes in a 5% CO_2_ incubator. CCF-STTG1 cells (also 3–4 passages from frozen seed stock) were cultured in RPMI 1640 with 10% FBS and 4 mM L-glutamine under the same conditions as SH-SY5Y cells. Each experiment involved 8 dishes per cell line. At 70–80% confluence, 4 dishes were moved to a 42°C incubator with 5% CO_2_ 1 hour before harvesting while 4 dishes were kept at the original 37°C incubator as controls. After the treatments, cells were harvested and extracted with detergents as described below.

### Sequential Detergent Extraction of the Proteins

**Figure 1:**
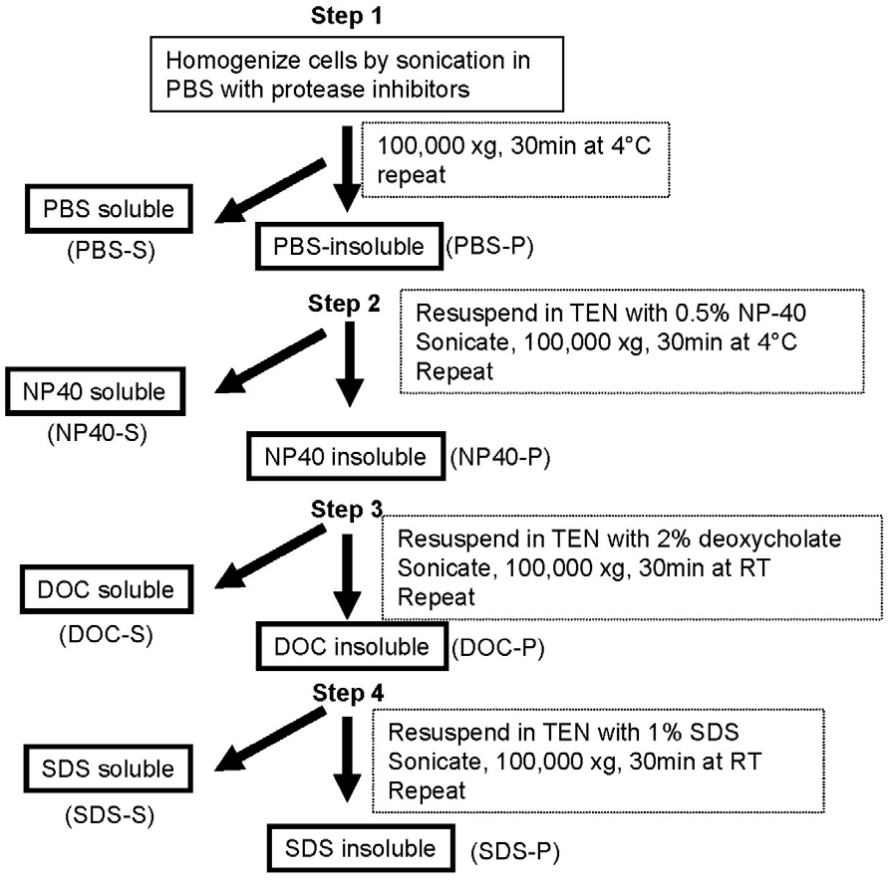
Diagram of detergent extraction and fractionation protocol.

The cells were harvested by scraping in PBS, and then sequential detergent extraction was used to fractionate the proteins according to their solubility (Figure 1). **Step 1)** The fresh cell pellet (cells from multiple dishes were combined to produce one cell pellet) was resuspended in 5 ml of PBS with 50 µl protease inhibitor cocktail (Sigma Aldrich, St. Louis, MO) before sonication on ice for 5–10 seconds [setting of 2 with an Ultrasonic Cell Disruptor (Microson, Newtown, CT)] to lyse the cells. The lysate was then centrifuged at 100,000×g for 30 minutes before the supernatant was collected and saved as the PBS-soluble (PBS-S) fraction. The pellet was washed by resuspension in 5 ml PBS (no protease inhibitor was used from this step forward) by brief sonication, followed by centrifugation at 100,000×g for 30 minutes at 4°C. The resultant supernatant was discarded. **Step 2)** The pellet was resuspended in 5 ml of TEN buffer (10 mM Tris-HCl, pH7.5, 1 mM EDTA and 100 mM NaCl) with 0.5% Nonidet-P40 (NP40) by brief sonication. An aliquot of this suspension was removed and saved as the PBS-insoluble (PBS-P) fraction. The remainder of the re-solubilized PBS-P fraction was centrifuged at 100,000×g for 30 minutes at 4°C. The supernatant was collected aliquoted and saved as the NP40-soluble fraction (NP40-S). The pellet was washed by resuspension in 5 ml of the same buffer by brief sonication before centrifugation at 100,000×g for 30 minutes. The resultant supernatant was discarded. **Step 3)** The pellet was resuspended in 5 ml TEN buffer with 2% sodium deoxycholate (DOC) by brief sonication. A aliquot of this suspension was removed and saved as the NP40-insoluble fraction (NP40-P) before the remaining suspension was centrifuged at 100,000×g for 30 minutes at room temperature. The supernatant was collected, aliquoted, and saved as the DOC-soluble (DOC-S) fraction. The pellet was washed by resuspension in 5 ml of the same buffer by brief sonication before centrifugation at 100,000×g for 30 minutes. The resultant supernatant was discarded. **Step 4)** The pellet was resuspended in 1 ml of TEN buffer with 1% sodium dodecyl sulfate (SDS) by brief sonication and 100–200 µl was removed and saved as the DOC-insoluble (DOC-P) fraction. To the remaining fraction, 4 ml of TEN with 1% SDS was added, mixed well, followed by centrifugation at 100,000×g for 30 minutes. The supernatant was collected as the SDS-soluble (SDS-S) fraction. The pellet after this extraction was very small. This sequential extraction procedure produced 9 samples: Total crude cell lysate, PBS-S (PBS soluble), NP40-S (NP-40 soluble), DOC-S (deoxycholate soluble), SDS-S (SDS soluble); PBS-P (PBS insoluble fraction), NP40-P (NP-40 insoluble fraction), DOC-P (deoxycholate insoluble fraction) and SDS-P (SDS insoluble).

### SDS-PAGE and Immunoblot

Proteins from different fractions were separated by 4–20% Tris-Glycine sodium dodecyl sulfate polyacrylamide gel electrophoresis (SDS-PAGE), and visualized by Coomassie blue staining. Parallel gels were run for immunoblot using antibodies against ubiquitin [5–25 (1∶5000), Signet] according to standard protocols. To validate the MS data ubiquitin antibodies [rabbit anti-ubiquitin (1∶1000), DAKO, MCA-UBi-1(1∶1000), Encor Biotech Inc. Gainesville, FL], CDK-1 antibody (rabbit polyclonal, 1∶500, Sigma), FEN-1 antibody (rabbit polyclonal, 1∶500, Sigma) and TDP-43 antibody (MCA-3H8, mouse monoclonal, 1∶5000, Encor Biotech. Inc. Gainesville, FL) were used according to standard protocols.

### Sample Preparation for Mass Spectrometry

From the Coomassie blue stained SDS-PAGE gel, each lane was separated out and then subsequently cut into 5–9 pieces, from the lowest to highest molecular weight. A total of 67 gel pieces were analyzed in experiment 1; and 48 gel pieces were analyzed in experiment 2. Standard in-gel trypsin digestion was used prior to LC-MS/MS protein identification following a protocol used by the Protein Chemistry Core (ICBR, University of Florida, Gainesville, FL). Briefly, gel pieces were diced into <1 mm^3^ cubes and washed with 50% acetonitrile/50 mM ammonium bicarbonate solution to remove SDS and Coomassie blue. Gel pieces were dried in a speedvac evaporator and rehydrated with 45 mM dithiothreitol (DTT)/50 mM ammonium bicarbonate for 30 minutes at 55°C to reduce disulfide bonds. This solution was subsequently replaced with freshly made 100 mM iodoacetamide/50 mM ammonium bicarbonate and incubated at room temperature for 30 minutes in the dark. The gel pieces were dried again, and then rehydrated with 10 ng/µl Trypsin (trypsin was suspended in 50 mM acetic acid at 1 µg/µl, then diluted in 50 mM NH_4_HCO_3_/10% acetonitrile)(Promega Co., Madison, WI) on ice for 1 hour, followed by incubation overnight at 37°C (16–20 hours). The reaction was stopped with 5% formic acid, or acetic acid, in 50% acetonitrile. Finally, the digested peptides were extracted in 50% acetonitrile and then 100% acetonitrile. Dried peptides were dissolved in 20 µl water with 0.1% formic acid before analysis by LC-MS/MS.

### Liquid Chromatography Tandem Mass Spectrometry (LC-MS/MS) Analysis

The digested peptides from all the samples in this study were analyzed with a hybrid quadrupole time-of-flight mass spectrometer (QSTAR® XL, Applied Biosystems, Foster City, CA). A 120 minute gradient from 5% acetonitrile to 40% acetonitrile was used for LC separation.

Tandem mass spectra were extracted by Analyst QS (version 1.1). Charge state deconvolution and deisotoping were not performed. All MS/MS samples were analyzed using Mascot (Matrix Science, London, UK; version 2.2.0) to search IPI databases [7] [IPI HUMAN (version 3.32, 67524 entries)] assuming digestion with trypsin. For bioinformatic analysis, we used X! Tandem (www.thegpm.org; version 2007.01.01.1) to search a subset of the IPI HUMAN database (version 3.80, 556 entries). Both Mascot and X! Tandem were searched with a fragment ion mass tolerance of 0.30 Da and a parent ion tolerance of 0.30 Da. Carbamidomethylation of cysteine was specified in Mascot and X! Tandem as a fixed modification. S-carbamoylmethylcysteine cyclization of the N-terminus, deamidation of asparagine and glutamine, oxidation of methionine, and ubiquitination of lysine were specified in Mascot and X! Tandem as variable modifications.

Scaffold (version 2_01_02, Proteome Software Inc., Portland, OR) was used to validate MS/MS based peptide and protein identifications. Later, data were converted and modified with a new version Scaffold (version 3_00_07). Peptide identifications were accepted if they could be established at greater than 95.0% probability as specified by the Peptide Prophet algorithm [8]. Protein identifications were accepted if they could be established at greater than 99.0% probability and contained at least 2 identified peptides. Protein probabilities were assigned by the Protein Prophet algorithm [9]. Proteins that contained similar peptides and could not be differentiated based on MS/MS analysis alone were grouped to satisfy the principles of parsimony. The false discovery rates at these probabilities are 0.1% on proteins and 5.3% on peptides.

### Data Compilation and Semi-quantification

The number of unweighted spectrum counts per protein was tabulated from the unfiltered Scaffold data for comparison. Unweighted spectrum counts from the different conditions were compared between samples; those proteins with a significant difference in the number of spectra were analyzed in more detail. To test whether the abundance of a particular protein is significantly higher in one sample than another, a spectral counting semi-quantification method was used [10]. The method for relative quantitation followed published protocols [11], [12] in which the change in abundance was determined by the ratio of the total number of identified MS/MS spectra (normalized unweighted spectral count from Scaffold) for a particular protein in the heat-shock treated and control groups respectively. A statistical G- test (likelihood ratio test for independence) was then utilized to determine the statistical probability that the abundance of a particular protein in a particular fraction was higher or lower than expected [11], [13]. To increase statistical power for G-test analysis of proteins identified with high-confidence (99% protein confidence, 95% peptide confidence and containing 2 unique peptides) we included in the data sets peptides with lower Mascot or Sequest scores that represent true positive identifications at 50% probability to match. All the spectra count numbers shown in this paper are based on 50% peptide probability for proteins that were identified at 95% confidence. Differences in protein composition between fractions were considered highly significant if the G-test significance was p<0.05.

### Gene Ontology and Other Bioinformatics Analysis on the Proteins

#### Protein theoretical isoelectric point (pI) and hydrophobicity

The pI and hydrophobicity of the whole human proteome (EBI database ipi.HUMAN.v.3.72) was calculated by Protein Digestion Simulator (version 2.0) developed by Department of Energy (PNNL, Richland, WA). Theoretical pI of proteins listed in Tables 1 and 2 were re-evaluated with the results calculated by Compute pI/Mw tool [Expert Protein Analysis System, ExPASy, of the Swiss Institute of Bioinformatics (SIB)] [14]. The pI from Protein Digestion Simulator is consistently 0.2–0.3 unit higher than calculated by ExPASy tool. Kyte-Doolittle scale, a widely used method for delineating hydrophobic character of a protein, was used to calculate the hydrophobicity of the proteins.

**Table 1:** Insoluble proteins in SH-SY5Y cells identified after 42°C heat-shock treatment.

Gene Symbol	Protein	Accession Number	Spectra count Exp 1 Exp 2				Range of p value byG-test
Group A			37	42	37	42	
**ASCC3L1**	Isoform 1 of U5 small nuclear ribonucleoprotein200 helicase	IPI00420014	0	8	1	23	2.8×10^−6^–0.007
**ANP32E**	Acidic leucine-rich nuclear phosphoprotein 32 family member E	IPI00165393 (+1)	0	8	0	5	0.007–0.047
**CDK1**	Cyclin-dependent kinase 1	IPI00026689	0	9	0	10	0.002–0.004
**CEBPZ**	CCAAT/enhancer-binding protein zeta	IPI00306723	2	11	0	5	0.016–0.047
**CHD4**	Isoform 1 of Chromodomain-helicase-DNA-bindingprotein 4	IPI00000846 (+1)	0	14	0	9	1.3×10^−4^–0.004
**DDX3X**	ATP-dependent RNA helicase DDX3X	IPI00215637	2	11	4	20	0.001–0.016
**EIF2AK2**	Interferon-induced, double-stranded RNA-activatedprotein kinase	IPI00019463	0	5	0	6	0.024–0.047
**FEN1**	Flap endonuclease 1	IPI00026215	0	8	0	6	0.007–0.024
**FTSJ3**	Putative rRNA methyltransferase 3	IPI00217686	1	7	0	6	0.024–0.050
**GTF2I**	Isoform 1 of General transcription factor II-I	IPI00054042 (+3)	3	17	2	32	6.3×10^−8^–0.002
**HNRPH1**	Heterogeneous nuclear ribonucleoprotein H	IPI00013881 (+1)	7	23	4	14	0.004–0.022
**HNRPH2**	Heterogeneous nuclear ribonucleoprotein H'	IPI00026230	4	15	0	9	0.003–0.014
**KIAA0020**	Pumilio domain-containing protein KIAA0020	IPI00791325	0	10	0	5	0.002–0.047
**KPNB1**	Importin subunit beta-1	IPI00001639	0	6	0	10	0.002–0.024
**KPNA2**	Importin subunit alpha-2	IPI00002214 (+1)	0	9	0	8	0.003–0.007
**MATR3**	Matrin-3	IPI00017297 (+1)	4	29	7	37	4.0×10^−6^–8.5×10^−6^
**MYBBP1A**	Isoform 1 of Myb-binding protein 1A	IPI00005024 (+1)	5	19	3	12	0.005–0.025
**PCBP2**	poly(rC)-binding protein 2 isoform b	IPI00012066 (+3)	0	6	0	6	0.024
**PDCD11**	RRP5 protein homolog	IPI00400922	5	18	3	21	2.1×10^−4^–0.008
**RPL7**	60S ribosomal protein L7	IPI00796861	0	13	0	9	2.5×10^−4^–0.004
**RRP12**	Isoform 1 of RRP12-like protein	IPI00101186 (+1)	0	12	0	6	4.8×10^−4^–0.024
**SND1**	Staphylococcal nuclease domain-containing protein 1	IPI00140420	0	11	0	24	1.5×10^−7^–9.3×10^−4^
**UBC**	Ubiquitin	IPI00783060 (+22)	1	19	3	19	3.6×10^−5^–6.4×10^−4^
**UBTF**	Isoform UBF1 of Nucleolar transcription factor 1	IPI00014533 (+1)	0	7	0	13	2.5×10^−4^–0.013
**UHRF1**	ubiquitin-like, containing PHD and RING finger domains,1 isoform 2	IPI00797279 (+1)	0	5	0	6	0.024–0.047
**Group B**			**37**	**42**	**37**	**42**	
**CRNKL1**	Isoform 1 of Crooked neck-like protein 1	IPI00177437 (+2)	0	4	0	5	0.047–0.088
**DDX27**	Probable ATP-dependent RNA helicase DDX27	IPI00293078	0	1	0	5	0.047–0.560
**DDX47**	Probable ATP-dependent RNA helicase DDX47	IPI00023972 (+1)	0	7	0	3	0.013–0.165
**MAGED2**	Isoform 1 of Melanoma-associated antigen D2	IPI00009542	0	2	0	5	0.047–0.306
**NOC4L**	Nucleolar complex protein 4 homolog	IPI00031661 (+1)	0	5	0	3	0.047–0.165
**NSUN2**	tRNA	IPI00306369	0	4	0	5	0.047–0.088
**PPM1G**	Protein phosphatase 1G	IPI00006167	0	6	0	3	0.024–0.165
**PRKDC**	Isoform 1 of DNA-dependent protein kinase catalytic subunit	IPI00296337	13	74	55	115	1.2×10^−11^–3.8×10^−6^
**RBM14**	Isoform 1 of RNA-binding protein 14	IPI00013174	0	4	0	9	0.003–0.088
**SUPT16H**	FACT complex subunit SPT16	IPI00026970	13	40	20	41	2.0×10^−4^–0.008
**TYMS**	Thymidylate synthase	IPI00103732 (+1)	0	5	0	2	0.047–0.306
**USP11**	ubiquitin specific protease 11	IPI00184533	0	3	0	5	0.047–0.165

**Table 2:** Insoluble proteins in CCF-STTG1 cells identified after 42°C heat-shock treatment.

Gene Symbol	Protein	Accession Number	Spectra count Exp 1 Exp 2				Range of p value by G-test
**Group A**			**37**	**42**	**37**	**42**	
**ANXA11**	Annexin A11	IPI00414320	0	10	0	10	0.002
**BYSL**	Bystin	IPI00328987	0	9	0	28	1.0×10^−8^–0.004
**CDK6**	Cell division protein kinase 6	IPI00023529	0	15	0	24	1.5×10^−7^–6.5×10^−5^
**HNRPH2**	Heterogeneous nuclear ribonucleoprotein H'	IPI00026230	0	6	1	12	0.003–0.025
**HNRPK**	Isoform 1 of Heterogeneous nuclear ribonucleoprotein K	IPI00216049 (+3)	0	15	0	12	6.5×10^−5^–4.8×10^−4^
**HSPB1**	Heat shock protein beta-1	IPI00025512	0	11	0	13	2.5×10^−4^–0.001
**MATR3**	Matrin-3	IPI00017297 (+1)	3	26	4	45	7.1×10^−10^–1.2×10^−5^
**RBM14**	Isoform 1 of RNA-binding protein 14	IPI00013174	0	6	0	6	0.024
**SND1**	Staphylococcal nuclease domain-containing protein 1	IPI00140420	0	16	0	36	4.4×10^−11^–3.3×10^−5^
**STAT1**	Isoform Alpha of Signal transducer and activator of transcription 1-alpha/beta	IPI00030781	0	8	0	15	6.5×10^−5^–0.007
**TTLL12**	Tubulin–tyrosine ligase-like protein 12	IPI00029048	0	5	0	5	0.047
**Group B**			**37**	**42**	**37**	**42**	
**ANXA5**	Annexin A5	IPI00329801	0	4	0	7	0.013–0.088
**CDK1**	Cyclin-dependent kinase 1	IPI00026689	0	3	0	11	9.3×10^−4^–0.165
**FEN1**	Flap endonuclease 1	IPI00026215	0	2	0	6	0.024–0.306
**FNDC3B**	Isoform 1 of Fibronectin type III domain-containing protein 3B	IPI00217490	0	2	0	7	0.013–0.306
**GTF2I**	Isoform 1 of General transcription factor II-I	IPI00054042 (+3)	0	2	0	23	3.0×10^−7^–0.306
**HDLBP**	Vigilin	IPI00022228	0	3	0	7	0.013–0.165
**HNRPA0**	Heterogeneous nuclear ribonucleoprotein A0	IPI00011913	0	4	0	6	0.024–0.088
**HNRPF**	Heterogeneous nuclear ribonucleoprotein F	IPI00003881	0	3	0	8	0.007–0.165
**HNRPH3**	Isoform 1 of Heterogeneous nuclear ribonucleoprotein H3	IPI00013877 (+1)	0	4	0	8	0.007–0.088
**HNRPM**	Isoform 1 of Heterogeneous nuclear ribonucleoprotein M	IPI00171903 (+1)	8	19	5	15	0.030–0.039
**HSPA8**	Isoform 1 of Heat shock cognate 71 protein	IPI00003865	0	14	7	16	1.3×10^−4^–0.069
**NONO**	Non-POU domain-containing octamer-binding protein	IPI00304596	0	3	0	6	0.024–0.165
**PCBP1**	Poly(rC)-binding protein 1	IPI00016610	0	2	0	5	0.047–0.306
**PCBP2**	poly(rC)-binding protein 2 isoform b	IPI00012066 (+3)	0	2	0	6	0.024–0.306
**RBM12B**	RNA binding motif protein 12B	IPI00217626	0	2	0	9	0.003–0.306
**TARDBP**	TDP43	IPI00025815	0	4	0	7	0.013–0.088
**TNS3**	Isoform 1 of Tensin-3	IPI00658152	0	4	0	19	4.4×10^−6^–0.088
**UBC**	Ubiquitin	IPI00783060 (+22)	7	16	6	28	1.5×10^−4^–0.069
**UBTF**	Isoform UBF1 of Nucleolar transcription factor 1	IPI00014533 (+1)	0	2	0	11	9.3×10^−4^–0.306
**ZC3HAV1**	Isoform 1 of Zinc finger CCCH type antiviral protein 1	IPI00410067 (+1)	0	1	0	6	0.024–0.560

#### Protein classification

Gene ontology analysis used PANTHER (Protein ANalysis THrough Evolutionary Relationships) software (http://www.pantherdb.org) [15] with the free online tools to classify genes and proteins by their functions, using published scientific experimental evidence and evolutionary relationships abstracted by curators with the goal of predicting function even in the absence of direct experimental evidence.

#### Protein relationship network

Pathway Studio software (version 7.1, Ariadne Genomics, Rockville, MD) was used to analyze the relationship between the identified insoluble proteins from Tables 1 and 2.

## Results

### Heat-shock Induces Intense Protein Ubiquitination and Aggregation in Cultured SH-SY5Y and CCF-STTG1 Cells

Cultured SH-SY5Y and CCF-STTG1 cells were incubated at 42°C for 1 hour before harvesting and sequential extraction to separate soluble and insoluble proteins (see Methods and Figure 1). To initially lyse the cells, we used a sonication approach that we have used repeatedly in prior studies to examine the misfolding of mutant superoxide dismutase 1 (SOD1) [16], [17]. Lysis by sonication provides a means to quickly lyse cells and shear nuclear DNA, which if left intact increases the viscosity of the solution and interferes with centrifugation sedimentation. The initial lysis was carried out in phosphate buffered saline (PBS) to first separate completely soluble proteins, followed by sequential extraction of insoluble fractions with nonionic detergent NP40, followed by the chaotropic agent deoxycholic acid (DOC), followed by the ionic detergent SDS. These detergents have been used to separate misfolded proteins such as mutant SOD1 [16] and huntingtin [18]. By sequentially extracting the preparation in these detergents of different strength, we sought to identify the detergent that provided the best differential between control and heat-shocked cells and produced the least complex sample.

**Figure 2:**
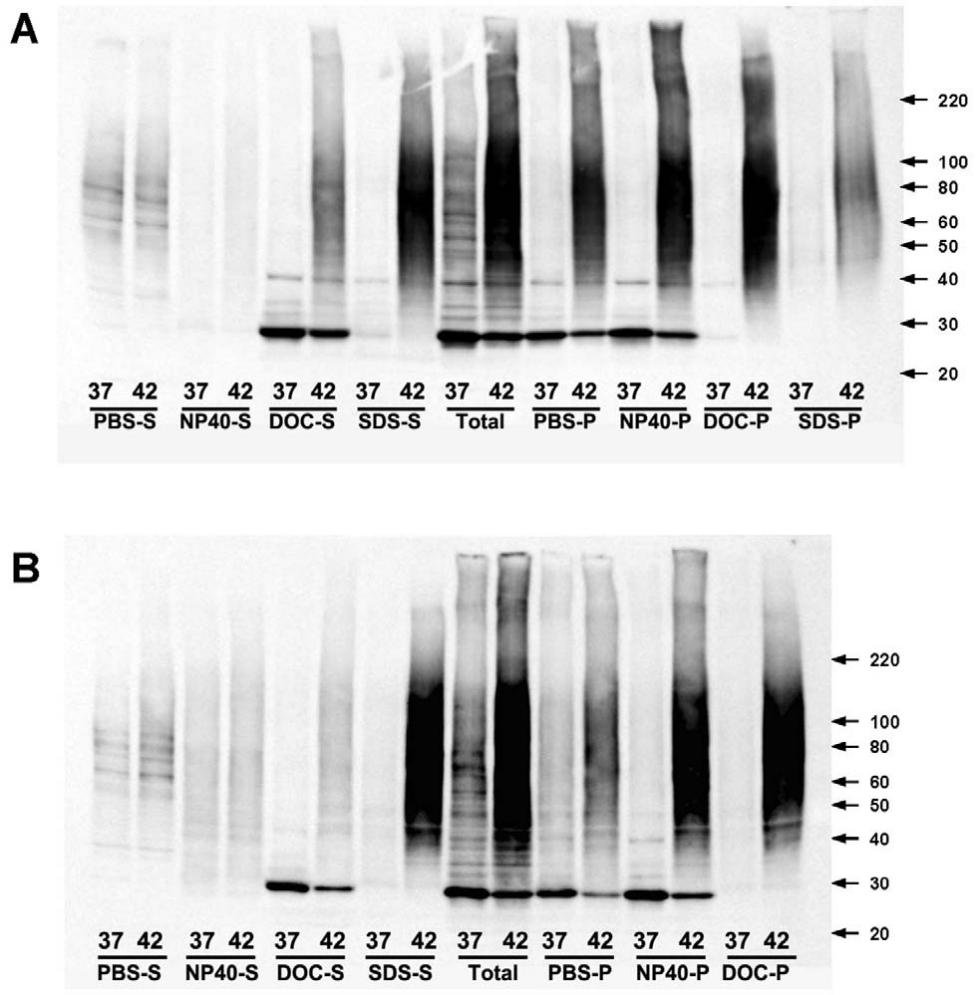
Accumulation of detergent-insoluble ubiquitinated proteins in heat-shocked SH-SY5Y (A) and STTG1 (B) cells. Immunoblot of cell fractions with antibodies to ubiquitin [1∶5000, (monoclonal 5–25, Signet, Covance Inc.)]. The image shown is representative of 3 repetitions of the experiment.

The fractionation procedure produced multiple fractions designated as the following; PBS-soluble fraction (PBS-S), PBS-insoluble (PBS-P), NP40-soluble (NP40-S), NP40-insoluble (NP40-), DOC-soluble (DOC-S), DOC-insoluble (DOC-P), SDS-soluble (SDS-S), and finally SDS-insoluble (SDS-P). Immunoblotting of these fractions with antibodies to ubiquitin (monoclonal 5–25, Signet, Covance Inc.) revealed that heat-shock induced a robust accumulation of insoluble polyubiquitinated proteins in both cell lines (Figure 2). The polyubiquitinated insoluble proteins were detected in the PBS-P, NP40-P, and DOC-P fractions. SDS solubilized most, but not all, of the proteins in the DOC-P fractions, leaving a relatively small amount of material in the SDS-P fraction (Fig. 2A) and even less in CCF-STTG1 cells (not shown). Thus, ultimately, the DOC-P fractions were identified as the least complex fractions that showed a difference between control and heat-shocked cells, and thus we chose to focus the LC-MS/MS analysis on these fractions. For comparison, we also chose to analyze the PBS-soluble fractions as we were interested in identifying those proteins that are normally soluble and then lose solubility upon heat-shock.

To determine the identity of proteins that lose solubility upon heat-shock, we used an approach in which soluble and insoluble fractions were further fractionated by SDS-PAGE with each lane of the gel subsequently cut into smaller pieces before in-gel digestion by trypsin and LC-MS/MS analysis (see Figure S1 for an example of SDS-PAGE). Using criteria described in Methods, a total of 651 proteins were identified in fractions from the SH-SY5Y cells, and 715 proteins in fractions from CCF-STTG1 cells at a protein false discovery rate of less than 0.1% (95% confidence in peptide identification) (Table S1). When we merged the Scaffold files of the two cell lines together, using the same criteria described above, we identified 965 proteins from these cells. 586 of the proteins were present in both cells lines, with 164 unique to SH-SY5Y cells and 215 unique to CCF-STTG1 cells (Figure S2).

We then used a G-test (see Methods) to compare unweighted spectra counts (essentially identified peptides) for each protein in DOC-P fractions from control and heat-shocked cells [11], [19] to identify those proteins that were significantly more abundant in DOC-P fractions after heat-shock. In those cases in which there were no spectra identified in the insoluble fraction of the control cells, the proteins met statistical significance in the G-test when we identified at least 5 spectra in the insoluble fraction of heat-shocked cells. Ubiquitin was the most abundant insoluble protein in both cell lines, with the highest sequence coverage (61%), and highest number of spectra per 100 amino acid (up to 28 unweighted spectra identified from this 76 amino acid protein) (Tables 1 and 2). These data are consistent with the immunoblots of these fractions, which detected intense ubiquitin immunostaining in the NP40-P and DOC-P fractions (Figure 2).

**Figure 3:**
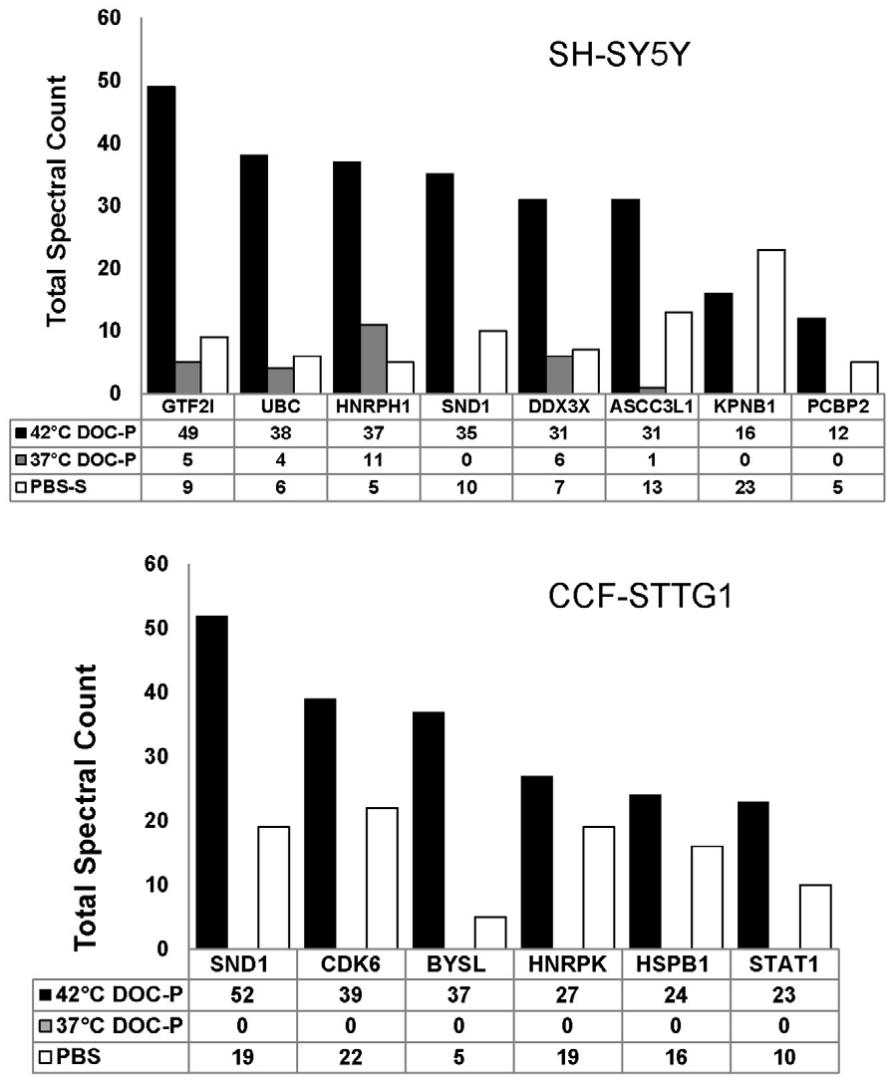
Spectral counts of proteins identified as over-represented in DOC-insoluble fractions of SH-SY5Y cells and CCF-STTG1 cells. Upper and lower panels, graphs spectral counts for proteins in SH-SY5Y and CCF-STTG1 cells, respectively, that were found in PBS-S as well as DOC-P fractions. Data were compiled from 2 separate proteomic analyses. The numbers graphed are sums of spectra numbers for the 2 experiments.

Using LC-MS/MS approaches, in human SH-SY5Y and CCF-STTG2 cells we found that, to varying degrees, a number of proteins showed increased numbers of peptide identifications in DOC-P fractions from heat-shocked cell lysates (Tables 1 and 2). In total, we identified 58 relatively abundant proteins, from the two cell lines, that showed consistent changes in solubility upon heat-shock. In analyzing the data, the proteins identified fell into 2 groups. Proteins in Group A showed the following characteristics: 1) showed >3-fold increase in spectral counts between control and heat-shocked cells in two experiments; 2) not detected in insoluble fractions of control cells and detected in insoluble fractions of heat-shocked cells (at >5 spectra in two experiments). In the SH-SY5Y cells, 25 proteins fit these criteria (Table 1– Group A). In CCF-STTG1 cells, 11 proteins fit these criteria (Table 2– Group A). Of the proteins we classified in Group A in both cell lines, many of these were also detected in PBS soluble fractions (Figure 3). Proteins placed in Group B met the above criteria in only one experiment. In SH-SY5Y cells, 12 proteins fit these criteria (Table 1- Group B) whereas in CCF-SSTG1 cells, 20 proteins fit these criteria (Table 2- Group B).

**Figure 4:**
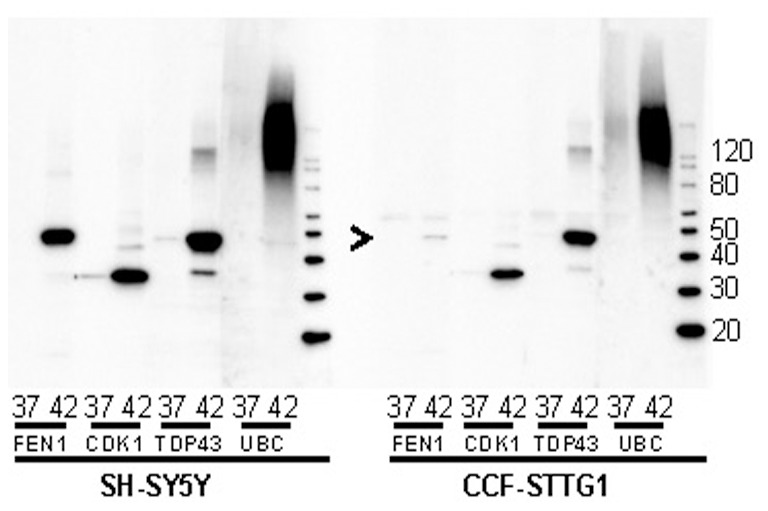
Immunoblot validation of LC-MS/MS data. Cells from each cell line were either held at 37°C or heat-shocked at 42°C and then lysed and fractionated as described in Methods. Equal amounts, by volume, of DOC-P fraction from control and heat-shocked cells were probed with antibodies to FEN1, CDK1, TDP-43, and Ubiquitin. The image shown is representative of 3 independent replications of the experiment.

Ideally, we would have liked to have validated the entire LC-MS/MS data set by immunoblotting of similarly prepared fractions. However, it proved challenging to identify specific high quality antibodies for most of the proteins. We were able to identify antibodies to 3 proteins in addition to ubiquitin that allowed us to spot validate the data set (Figure 4). Commercially available antibodies to FEN1, CDK1, and TDP-43 were identified as showing good reactivity and identification of a band of the expected size. In SH-SY5Y cells, FEN1 and CDK1 were examples of proteins we classified in Group A, and both of these validated (Figure 4). TDP-43 was identified as over-represented in insoluble fractions from CCF-STTG1 cells but did not meet in either experiment with SH-SY5Y cells. Immunoblot data, however, clearly showed that this protein loses solubility upon heat-shock in both cell lines. As expected, ubiquitin immunoreactivity was more abundant in the insoluble fractions from heat-shocked cells. All three of these proteins were identified in the LC-MS/MS data set in CCF-STGG1 cells as potentially showing shifts in solubility after heat shock and all three were validated by immunoblotting (Figure 4). The levels of FEN1 were low in the insoluble fractions from these cells, but a band of a size similar to what was detected in SH-SY5Y cells was observed (Figure 4, arrowhead). Although these data do not validate the entire set of candidates, the data do indicate that the LC-MS/MS data provide good predictability regarding protein sensitivity to thermal stress.

**Figure 5:**
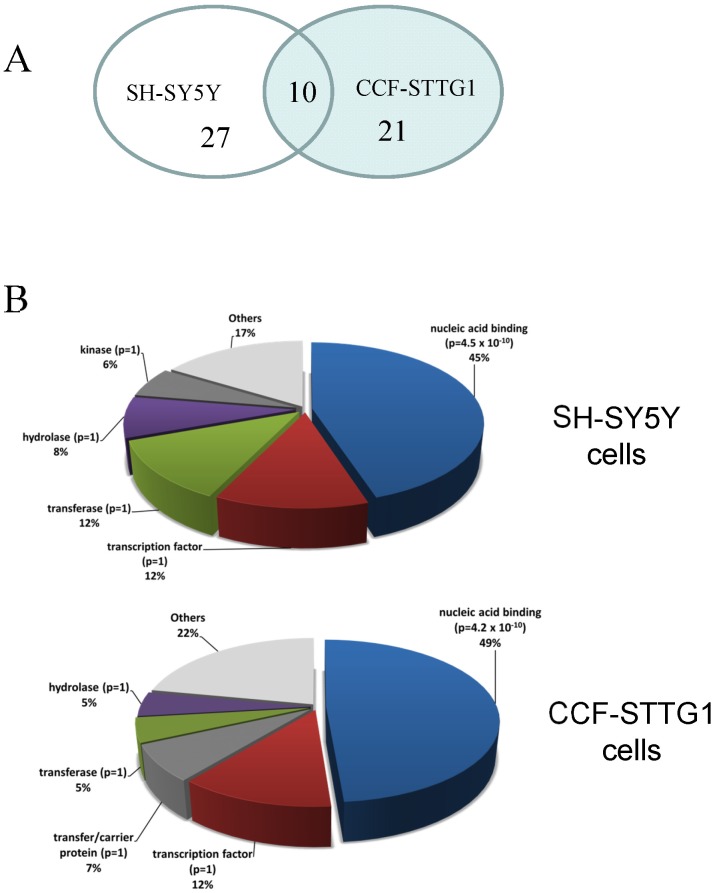
Bioinformatic analysis of LC-MS/MS data. A) Comparison of proteins that were over-represented in DOC insoluble fractions rom both cell lines. Only 11 proteins were common to the DOC insoluble fractions from both cell lines. B) Gene ontology analysis. Online Panther analysis tools 7.1 were used to determine the protein classes of the proteins listed in Tables 1 and 2. Only the proteins identified as significantly more abundant in the insoluble fractions from both experiments were used for this analysis.

In comparing the proteins identified in each cell type as showing diminished solubility after heat-shock by LC-MS/MS, we found limited overlap between the two cell lines. Of the 58 unique proteins identified in the 2 cell lines, only 10 were common between the 2 cell lines (Figure 5A). Among the proteins that fit criteria for Group (in Tables 1 and 2), HNRPH2, MATR3, and SND1 were the only proteins found to shift solubility in both cell lines in both experiments. Seven additional proteins that were shared between the cell lines were identified as specifically losing solubility in only one of the two experiments.

Gene ontology analyses of the insoluble proteins from the heat-shocked cells, using Panther tools (http://www.pantherdb.org/) [15], [20], determined that most could be associated with known GO terms for biologic processes, cellular components and molecular functions. Although the identities of the proteins that were over-represented in the DOC-P fractions from heat-shocked cells of both cell lines differed, Panther protein classification demonstrated that the proteins listed in Tables 1 and 2 were primarily nuclear proteins that are involved in nucleic acid binding functions (Figure S2; Figure 5B). Nuclear proteins were the only class of proteins that were statistically over-represented in the data from both cell lines (Figure 5B).

**Figure 6:**
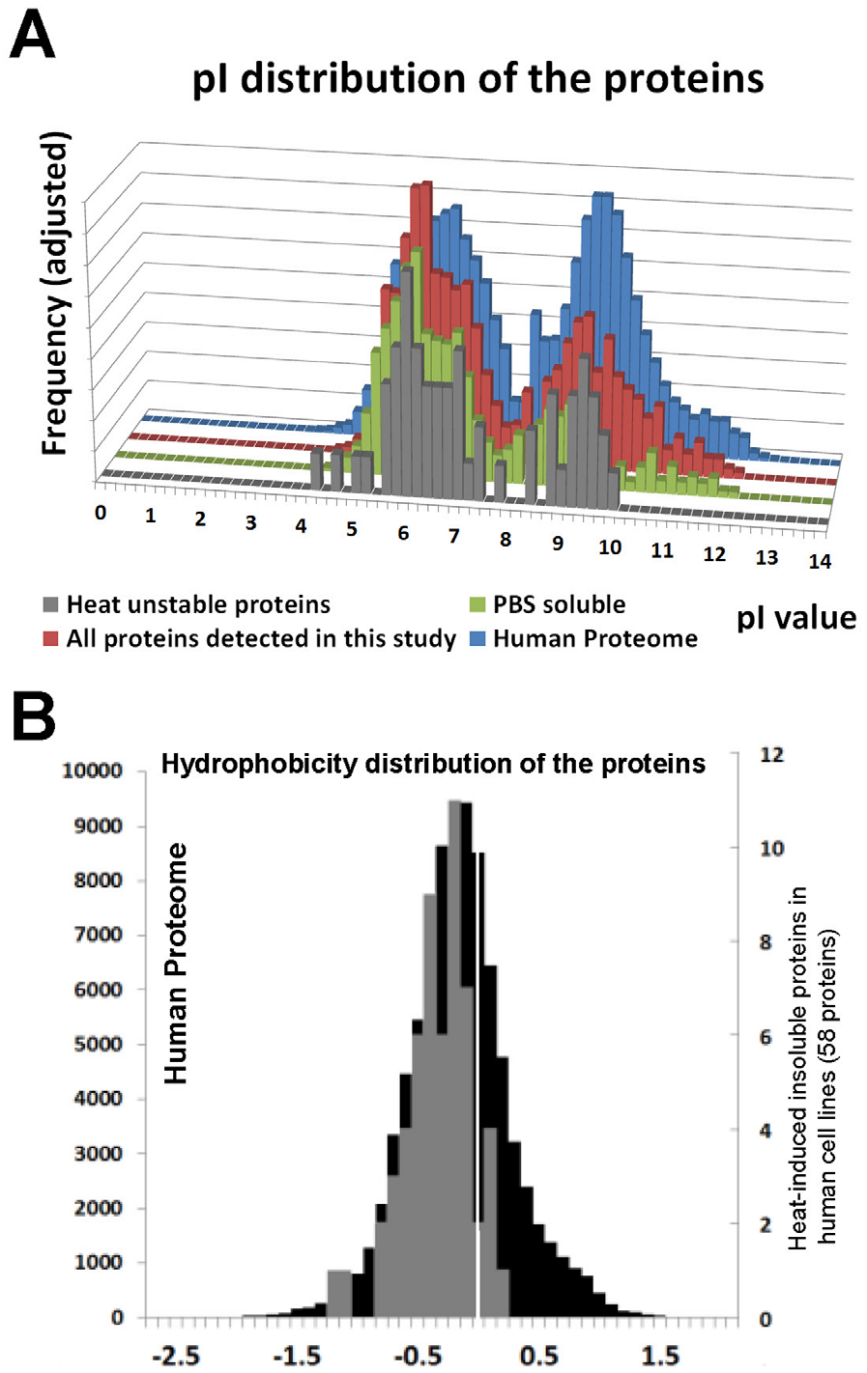
Comparisons of physiochemical characteristics of the proteins identified to be insoluble after heat-shock treatment in SH-SY5Y and CCF-STTG1 cell lines. (A) Protein theoretical isoelectric point (pI), and (B) hydrophobicity, between the heat-shock induced insoluble proteins identified from this study with the whole human proteome. For pIs, all the proteins identified in LC-MS/MS data in any fraction and all the soluble proteins identified in LC-MS/MS data were also included in comparison. Predicted pI values were obtained from the IPI human protein database (version 3.72) and the pI distribution was plotted with 0.2 pH unit increments. Hydrophobicity values (Kyte-Doolittle scale, the white mid-line is 0.) were also obtained from the same database and were plotted with 0.1 unit increment. In (B) the black bars graph data from the whole proteome and the gray bars graph data from the insoluble proteins of the heat-shocked cells.

To further analyze the data, we asked whether the proteins that lose solubility upon heat-shock possess distinct charge or hydrophobicity characteristics. The theoretical protein pI and hydrophobicity of the whole human proteome (EBI database ipi.HUMAN.v3.72 from European Bioinformatics Institute) was calculated by Protein Digestion Simulator (version 2.0). One of the most important determinants for a particular protein’s solubility is its electrostatic charge, which is governed by its amino acid sequence and the pH of aqueous solvent it is dissolved in. When net charge approaches zero, interactions between protein molecules are fostered rather than between protein and water molecules, leading to aggregation or insolubility [21]. We found that the pI distribution of the metastable proteins in SH-SY5Y and CCF-STTG1 cells was not obviously different from that of whole human proteome, nor from the total proteins or soluble proteins we identified in our LC-MS/MS data from both cell lines (Figure 6A). The hydrophobicity of the proteins was calculated using the Kyte-Doolittle scale, a widely used method for delineating hydrophobic character of a protein. Regions with values above 0 are hydrophobic. We found the distribution of the hydrophobicity of these proteins was also similar to that of the whole human proteome (Figure 6B), meaning that the heat-sensitive proteins were not particularly hydrophobic. Thus, neither charge nor hydrophobicity characteristics of these proteins made them more likely to become insoluble after heat-shock treatment.

### Polyubiquitin Linked by Conjugation at K-48 Accumulates in Heat-shocked Cells

In both heat-shock cell lines, we detected a robust accumulation of polyubiquitin by immunoblotting. To characterize these polyubiquitin chains, we used Mascot and X!Tandem algorithms to search for peptide motifs of ubiquitin-conjugated proteins. After trypsin digestion, a ubiquitin-conjugated protein contains a diglycine remnant of ubiquitin covalently attached to a lysine residue that remains after trypsin proteolysis. Cleavage at the peptide backbone results in a characteristic mass shift of +114 Da (Gly-Gly) on fragment ions containing ubiquitinated lysine residues after trypsin digestion.

**Figure 7:**
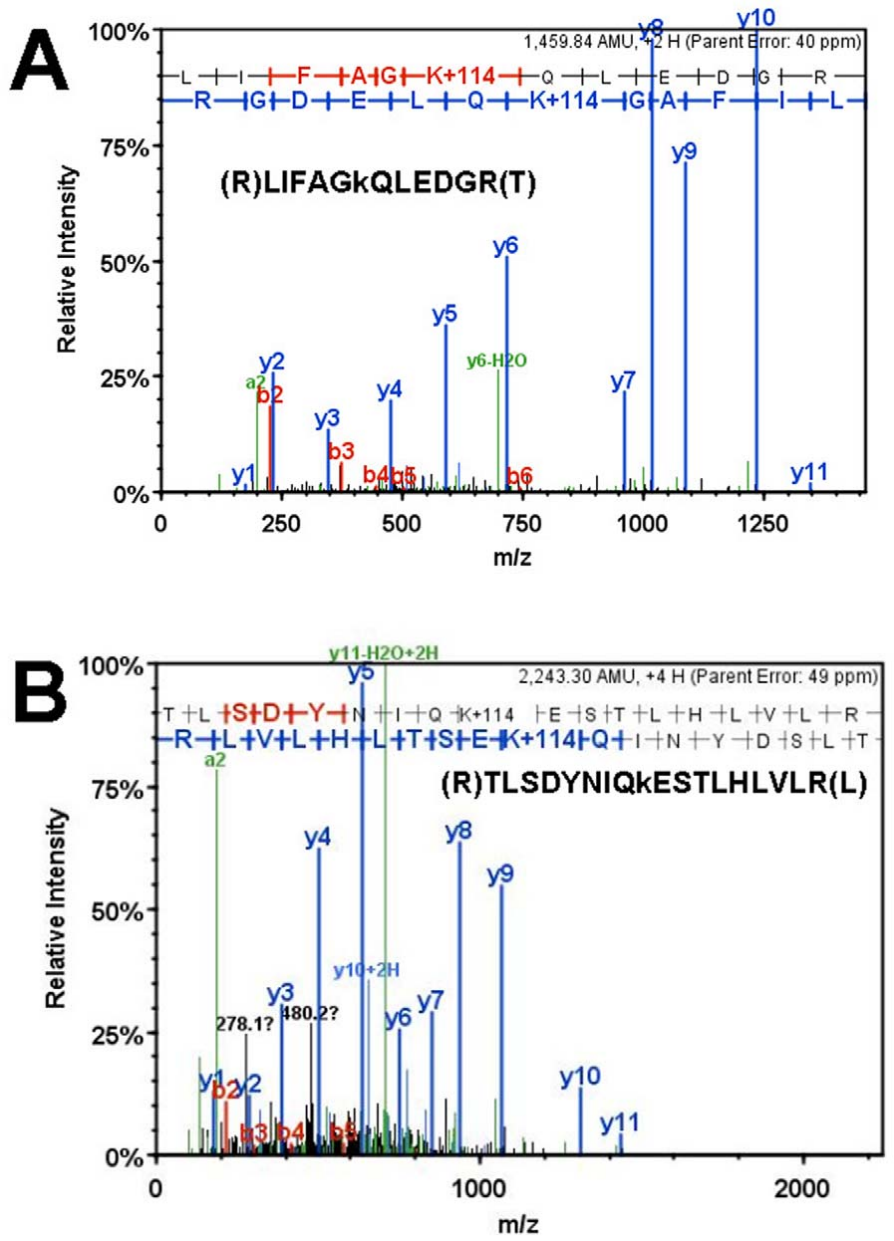
Representative spectrum of polyubiquitin linkage signatures for K-48 ubiquitin conjugation (A) and K-63 ubiquitin conjugation(B). Mass K+114 is the marker of ubiquitination (K-GlyGly modification). These spectra were abundant in both proteomic analyses.

From the DOC insoluble fractions of the cell heat-shock cells, we found relatively few proteins that possessed ubiquitinated peptides. By combining all the data generated from the 2 cell lines in Scaffold, only ubiquitin, Histone H3.1, actin and kynureninase were identified to contain K-GlyGly modified peptides among all 965 proteins. The most abundant peptide possessing the K-GlyGly modification was ubiquitin itself (42 out of 49 spectra). Ubiquitinated-ubiquitin peptides were found in 33 gel pieces, corresponding to various molecular weight ranges. Most of ubiquitin was linked to itself by conjugation at Lys-48 to form polyubiquitin chains, but there was also significant ubiquitination at Lys-63 (Figure 7).

## Discussion

In the present study, we have used proteomic approaches in conjunction with heat-shock of neural cell lines to identify proteins that are sensitive to thermal denaturation. In LC-MS/MS analysis we identified 58 candidate proteins of which 3 along with ubiquitin were validated by immunoblotting. The 58 candidate proteins were not distinct in regard to pI or hydrophobicity. Although the LC-MS/MS generated lists of proteins that populated the detergent insoluble fractions from heat-shocked cells of both cell lines were relatively distinct, the differences in protein identifications between the cells may be due largely to sampling errors in the mass spectrometry. For example, the spectral counts for TDP-43 in insoluble fractions from heat-shocked cells met statistical criteria only in CCF-STTG1 cells. Yet, immunoblots of insoluble fractions from the SH-SY5Y cells, as well as CCF-STTG1 cells, demonstrated that TDP-43 loses solubility upon heat-shock. Thus, there may be less differences between SH-SY5Y cells and CCF-STTG1 than implicated by the LC-MS/MS data. It is likely that if we could validate each candidate identified from the LC-MS/MS data by immunoblot, we would find that there are relatively few differences between the cell lines.

In viewing the LC-MS/MS data, we viewed the data as breaking into two groups based on relative abundance in insoluble fractions from control and heat-shocked cells, and whether the data for a given protein met criteria in both replicate experiments. Partially out of necessity, we focused on proteins in which we could identify at least 5 spectra in the insoluble fraction from the heat-shocked cells when the spectral count for the same fraction in control cells was zero because spectral counts below 5 typically did not reach statistical significance in the G-test and therefore were viewed as less reliable. Obviously the data in which we did not observe statistically significant differences in two experiments (Group B) were viewed as less reliable.

Validation of the entire LC-MS/MS data set for both cell lines by immunoblot was not feasible due to a lack of availability of validated high quality antibodies. We were able to accomplish what would be considered spot validation using four antibodies that recognized FEN1, CDK1, TDP-43, and ubiquitin. Ubiquitin is an example of a protein that mass spectrometry data ranked in Group A in SH-SY5Y cells and in Group B in CCF-STTG1 cells. Immunoblot validation of ubiquitin showed very robust signals in the detergent-insoluble fractions of heat-shocked cells from both cell lines. FEN1 and CDK1 were classified in Group A in SH-SY5Y cells and in Group B in CCF-STTG1 cells. Again, immunoblot analysis of insoluble fractions from these cells demonstrated selective loss in solubility upon heat shock in both cell lines. As mentioned above TADRBP (TDP-43) is an example of a protein that was in Group B in the CCF-STTG1 cells and scored as non-significant in SH-SY5Y cells. We note in in SH-SY5Y cells the LC-MS/MS data were 0 and 3 spectra experiment 1, and 2 and 4 spectra experiment 2, for control and heat-shocked cells respectively. Thus, although the data for SH-SY5Y cells were in the right direction, the data did not meet statistical criteria for significance.

Because of the inherent problems with LC-MS/MS sampling, the list of proteins we identify as sensitive to thermal denaturation in these cell lines should not be viewed as comprehensive or all inclusive, but rather as candidate. We were not able to adequately sample proteins of low abundance because a sufficient number of spectra were not detected to make accurate quantification. Additionally, although this initial list of proteins that lose solubility upon heat shock is heavily populated by nuclear proteins involved in transcription and RNA metabolism, bias in LC-MS/MS sampling or introduced our fractionation method may produce a false view of which proteins are most vulnerable.

### Heat-shock as a Model System to Identify Metastable Proteins

Metastable proteins are defined as entities that are inherently less able to maintain native conformation at physiological temperatures [2]. The protein homeostasis network, which includes protein chaperones, the ubiquitin/proteasome system, the autophagic system, and the protein synthesis machinery, functions in balance to maintain the proteome. Heat-shock produces an acute disturbance to this system and cells respond to this insult by inducing expression of chaperones while simultaneously shutting down new protein synthesis and up-regulating ubiquitination and degradation [22], [23]. Although heat-shock is clearly an acute insult to the proteome, this paradigm provides a model system to identify potentially metastable proteins as we would predict that such proteins would be more sensitive to thermal stress. The LC-MS/MS data reported here provides a lengthy list of candidate metastable proteins. We definitively identify FEN1, CDK1, and TDP-43 as being sensitive to thermal stress and thus potentially are metastable proteins. It is likely that there are many others that will ultimately be identified.

Among these three proteins, TDP-43 is an interesting potentially metastable proteins for several reasons. TDP-43 was first identified is a component of inclusion pathology found in individuals with Fronto-temporal dementia [24] and sporadic ALS [24]. TDP-43 immunoreactive inclusion pathology has also now been reported in patients with Alzheimer’s disease, Pick’s disease and Huntington’s disease [25]–[27]. Our identification of TDP-43 as a protein that is sensitive to thermal stress may provide an explanation for why this protein could appear as a pathological feature of so many different neurodegenerative diseases.

In addition to the proteins that we identify that lose solubility; the accumulation of K-48 linked polyubiquitin is a reliable biomarker of disturbed proteostasis. In the heat-shock model we use here, we are confident that the accumulation of K-48 linked polyubiquitin is a consequence of a sudden increase in proteasome substrates. However, it is also clear that inhibition of the proteasome can induce the accumulation of K-48 linked polyubiquitin [28]. In addition to polyubiquitin linked by K-48 conjugation, we also found ubiquitin conjugated at the K-63 site. Ubiquitin linked by K-63 linkage has been implicated in autophagic clearance of protein inclusions [29] and we assume that the autophagic system has also been transiently over-whelmed in heat-shocked cells.

### Conclusions

In the present study, we have sought to identify proteins that are sensitive to thermal denaturation. FEN1, CDK1, and TDP-43 were definitively identified as being sensitive to thermal stress. These proteins may represent natural metastable proteins of the human proteome. The appearance of insoluble forms of these proteins in conjunction with the accumulation of polyubiquitin chains linked through conjugation at lysine 48 may be a useful means to detect disturbances in protein homeostasis in mammalian cells.

## Supporting Information

Figure S1 **Coomassie Blue stained gels of total protein from the various fractions from each cell line.** (A) SH-SY5Y; (B) CCF-STTG1. From left to right: PBS-soluble, DOC-insoluble of control cells (37°C), and DOC-insoluble of heat-shock treated cells (42°C). Each lane was loaded with 45 µl of each fraction; two duplicate lanes were loaded per sample. These gels are representative of the gels used to generate the proteomic data.
(TIF)

Figure S2 **Venn diagram depicting overlap between the proteins identified from two cell lines.**
(TIF)

Figure S3 **Location and interaction network of detergent insoluble proteins identified in SH-SY5Y (A) and CCF-STTG1 (B) cells.** Proteins listed in Tables 1 and 2 were used to build this interaction network. Pathway Studio 7.2 was used which automatically mines data from scientific literature in PubMed. This network was built including common upstream regulators, common downstream targets and the direct interaction between these proteins. Cell processes are shown as yellow rectangles. The linkages with fewer than 5 references were removed.
(TIF)

Table S1 **Excel files of peptide spectra data.** There are 4 sheets to the file named Merged SH-SY5Y and STTG1 (all identified proteins in both cell lines), STTG1 (proteins identified only in STTG-1 cells, SH-SY5Y (proteins identified only in SH-SY5Y cells), and Merged PBS-S (proteins identified in the PBS-S fraction from both cell lines) that tabulate the spectra counts for the proteins identified in this study.
(XLSX)
