# Correction: Identification of Proteins Sensitive to Thermal Stress in Human Neuroblastoma and Glioma Cell Lines

**DOI:** 10.1371/annotation/82b96c01-6435-4856-80a6-0176b1986e32

**Published:** 2012-11-26

**Authors:** Guilian Xu, Stanley M. Stevens, Firas Kobiessy, Hilda Brown, Scott McClung, Mark S. Gold, David R. Borchelt

The third author's name was spelled incorrectly. The correct name is: Firas Kobeissy.

The correct citation is: Xu G, Stevens SM Jr, Kobeissy F, Brown H, McClung S, et al. (2012) Identification of Proteins Sensitive to Thermal Stress in Human Neuroblastoma and Glioma Cell Lines. PLoS ONE 7(11): e49021. doi:10.1371/journal.pone.0049021

