# Supplementary figures and images for: Identification of Proteins Sensitive to Thermal Stress in Human Neuroblastoma and Glioma Cell Lines
Source: PLoS One. 2012 Nov 8;7(11):e49021. doi: 10.1371/journal.pone.0049021 (PMC3493505; doi:10.1371/journal.pone.0049021)

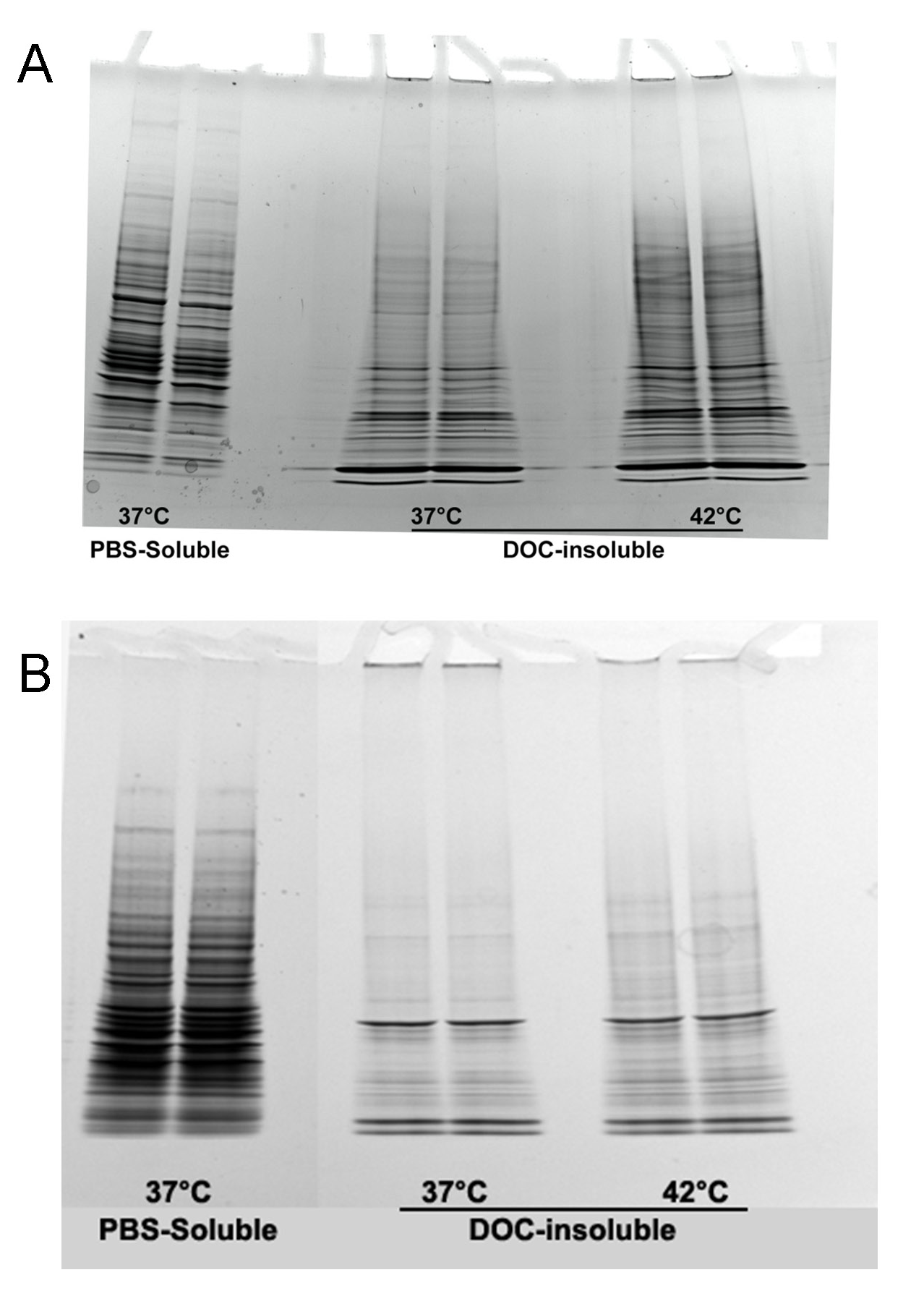

Supplement: Figure S1 — Coomassie Blue stained gels of total protein from the various fractions from each cell line. (A) SH-SY5Y; (B) CCF-STTG1. From left to right: PBS-soluble, DOC-insoluble of control cells (37°C), and DOC-insoluble of heat-shock treated cells (42°C). Each lane was loaded with 45 µl of each fraction; two duplicate lanes were loaded per sample. These gels are representative of the gels used to generate the proteomic data. (TIF) [file pone.0049021.s001.tif]

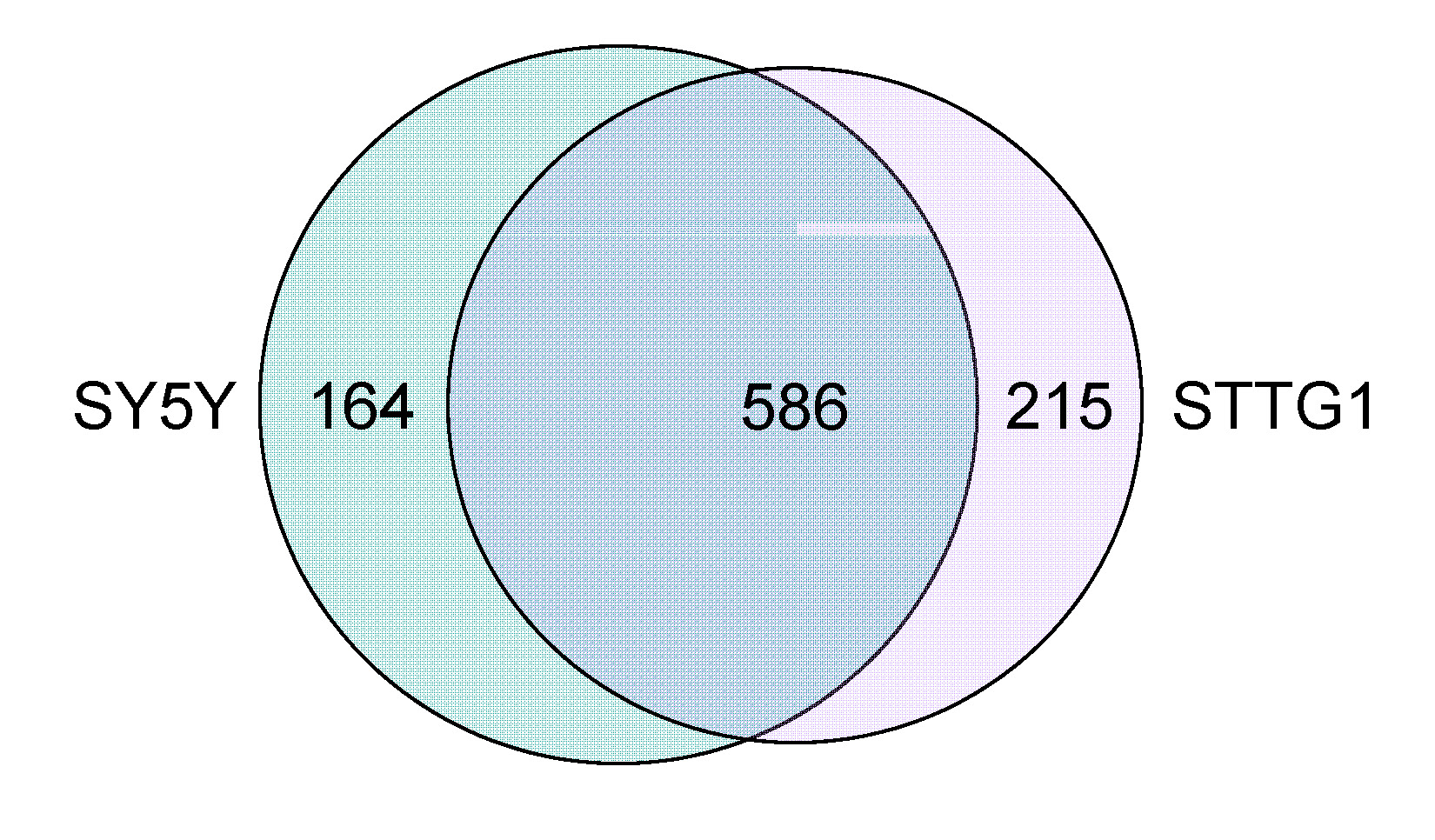

Supplement: Figure S2 — Venn diagram depicting overlap between the proteins identified from two cell lines. (TIF) [file pone.0049021.s002.tif]

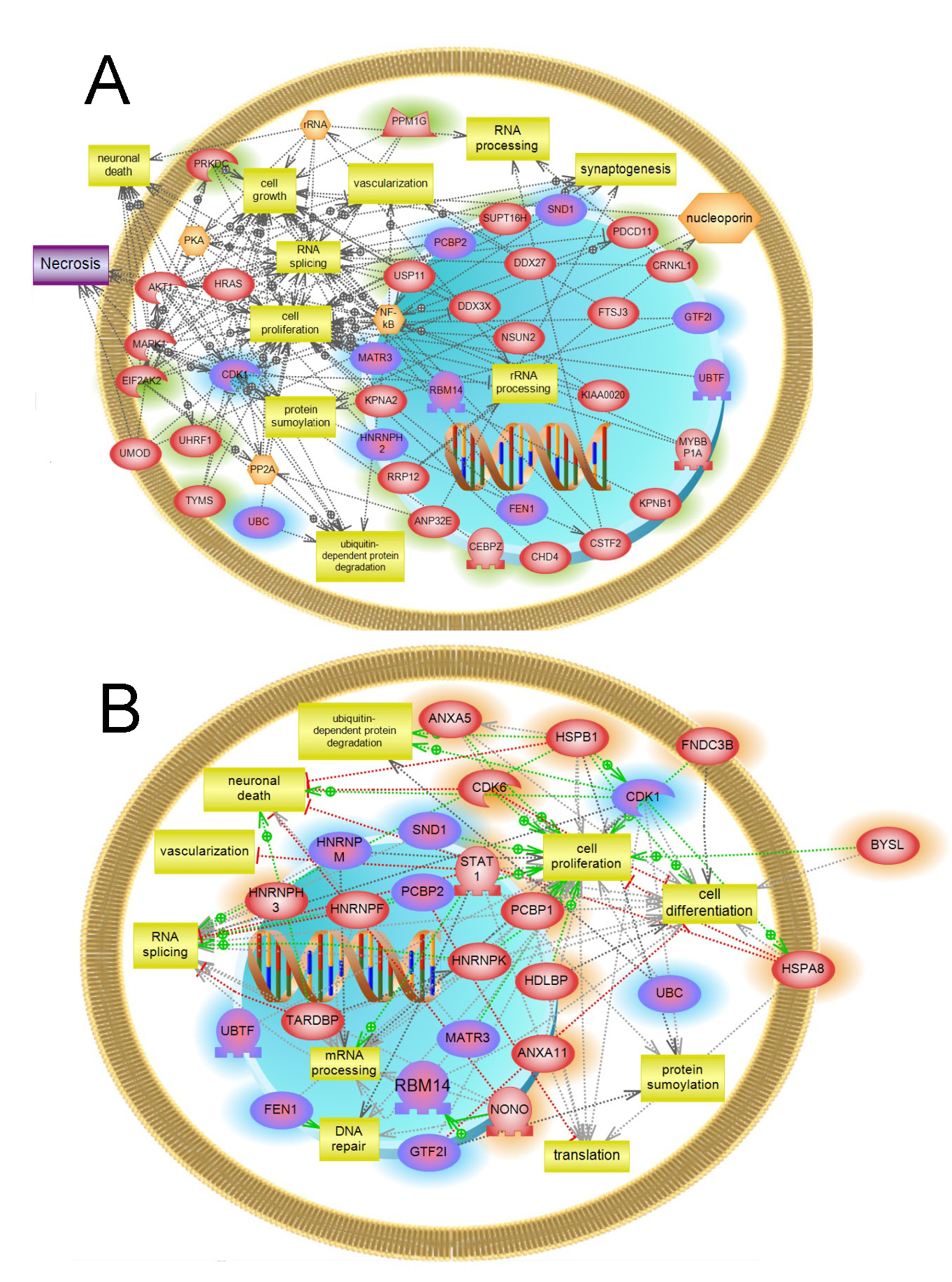

Supplement: Figure S3 — Location and interaction network of detergent insoluble proteins identified in SH-SY5Y (A) and CCF-STTG1 (B) cells. Proteins listed in Tables 1 and 2 were used to build this interaction network. Pathway Studio 7.2 was used which automatically mines data from scientific literature in PubMed. This network was built including common upstream regulators, common downstream targets and the direct interaction between these proteins. Cell processes are shown as yellow rectangles. The linkages with fewer than 5 references were removed. (TIF) [file pone.0049021.s003.tif]
